# Metal-Organic Framework (MOF)-Based Biomaterials for Tissue Engineering and Regenerative Medicine

**DOI:** 10.3389/fbioe.2021.603608

**Published:** 2021-03-11

**Authors:** Moldir Shyngys, Jia Ren, Xiaoqi Liang, Jiechen Miao, Anna Blocki, Sebastian Beyer

**Affiliations:** ^1^Department of Biomedical Engineering, The Chinese University of Hong Kong, Shatin, Hong Kong; ^2^Institute for Tissue Engineering & Regenerative Medicine, The Chinese University of Hong Kong, Shatin, Hong Kong; ^3^School of Biomedical Sciences, Faculty of Medicine, The Chinese University of Hong Kong, Shatin, Hong Kong; ^4^Department of Orthopaedics & Traumatology, Faculty of Medicine, The Chinese University of Hong Kong, Shatin, Hong Kong

**Keywords:** metal-organic frameworks, biomaterial, tissue engineering, regenerative medicine, bone, cardio-vascular, nervous tissue

## Abstract

The synthesis of Metal-organic Frameworks (MOFs) and their evaluation for various applications is one of the largest research areas within materials sciences and chemistry. Here, the use of MOFs in biomaterials and implants is summarized as narrative review addressing primarely the Tissue Engineering and Regenerative Medicine (TERM) community. Focus is given on MOFs as bioactive component to aid tissue engineering and to augment clinically established or future therapies in regenerative medicine. A summary of synthesis methods suitable for TERM laboratories and key properties of MOFs relevant to biomaterials is provided. The use of MOFs is categorized according to their targeted organ (bone, cardio-vascular, skin and nervous tissue) and whether the MOFs are used as intrinsically bioactive material or as drug delivery vehicle. Further distinction between *in vitro* and *in vivo* studies provides a clear assessment of literature on the current progress of MOF based biomaterials. Although the present review is narrative in nature, systematic literature analysis has been performed, allowing a concise overview of this emerging research direction till the point of writing. While a number of excellent studies have been published, future studies will need to clearly highlight the safety and added value of MOFs compared to established materials for clinical TERM applications. The scope of the present review is clearly delimited from the general ‘biomedical application’ of MOFs that focuses mainly on drug delivery or diagnostic applications not involving aspects of tissue healing or better implant integration.

## Introduction

MOFs are new synthetic materials that emerged over the past three decades (Yaghi and Li, 1995; Yaghi et al., 1995). MOFs comprise of organic ligands that bridge metal ions. This leads to highly ordered, porous and 3-dimensional crystalline structures. The pore size and aperture is significantly smaller compared to polymeric structures (Pan et al., 2013) and can be accurately controlled (Eddaoudi et al., 2002; Banerjee et al., 2009). This accurate control makes MOFs predestined for drug loading and sustained or triggered release from nano-particulate drug formulations or coatings (Wang et al., 2020). This has led to the classical understanding of “biomedical MOF applications” that primarily focused on diagnostic applications (Chapartegui-Arias et al., 2019) or drug delivery (Keskin and Kizilel, 2011; Banerjee et al., 2020). The present review is clearly delimited from these established fields of investigation by focusing on MOFs for TERM applications. In particular, the present review addresses MOFs that modulate the foreign body response to implants, MOFs that influence the inflammatory wound environment, or MOFs that improve the host tissue-implant interface and aid healing.

A systematic literature analysis was performed using Clarivate Analytics Web of Science database in combination with a structured key word search. In brief, a comprehensive list of works on MOFs was generated and those interfacing with biomaterials, tissue engineering, regenerative medicine or MOFs interfacing with neuronal repair were identified. Works solely focusing on drug-delivery or cancer treatment were excluded if not reporting aspect of tissue healing, integration or improvement of the foreign body response.

**FIGURE 1:**
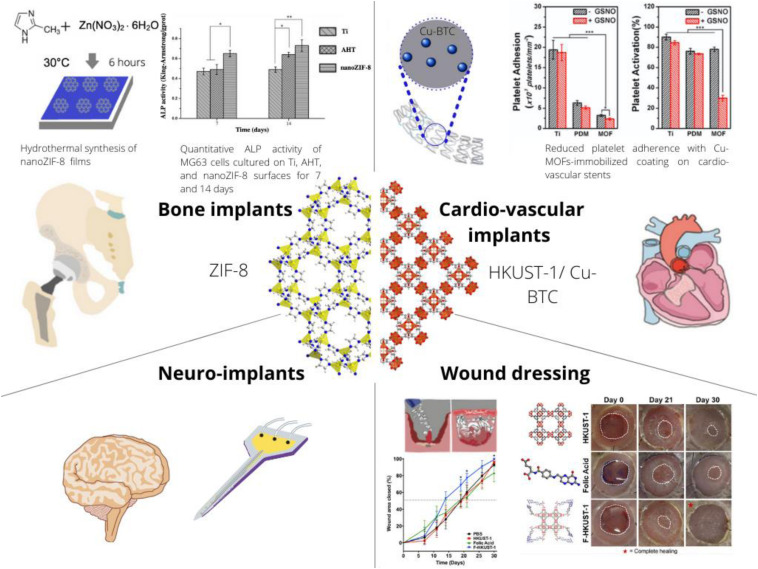
Schematic on the present and envisioned use of MOFs as a component of implants or biomaterial compositions with applications in Tissue Engineering and Regenerative Medicine (TERM). The crystal structure of the prototypical MOF Zeolitic Imidazolate Framework 8 (ZIF-8) is depicted along the [111] crystallographic direction (left side) and HKUST-1 also known as Cu-BTC is depicted along the [001] crystallographic direction. These two different structures have been most frequently applied to TERM research and represent the diverse class of MOFs. In particular, simple exposure of osteoblast like cells to ZIF-8 increases ALP activity (Chen et al., 2017a), Cu-BTC can decrease adherence and activation of platelets (Fan et al., 2019) and also accelerates skin wound closure (Xiao et al., 2018).

In addition to the drug loading capacity, the present review aims to highlight that MOFs may also have intrinsic bioactivity through delivering specific metal ions (Su et al., 2019), ligands (Rojas et al., 2017) or through catalytically generating messenger molecules such as nitric oxide in copper-based MOFs (Harding and Reynolds, 2012). Beyond individual components, structural aspects of MOFs are essential since they determine release kinetics as well as the accessibility of the metal centers providing catalytic effects. Most studies investigating MOFs for TERM application focused on orthopedic applications, followed by cardiovascular treatment, cutaneous wound healing, and applications in nervous tissue (Figure 1). Beyond these targeted organs, a specialized MOF was proposed for treatment of periodontitis (Li et al., 2019), while materials for other organs seem not yet to have been evaluated. In summary, the use of MOFs for TERM applications is an emerging field of research with only very few works published before the year 2018.

### Parallels of MOFs to Natural Minerals in TERM Applications

The concept of MOFs originates from natural minerals, which have a substantial track record in TERM applications. Calcium phosphate has intrinsic osteogenic effect (Albee, 1920) and its admixtures to biodegradable polymers led to clinical implants (de Groot, 1993). A calcium and strontium based MOF with bisphosphonate linker (Matlinska et al., 2019) appears to be the evolution of minerals with engineered bioactivity.

Another naturally occurring mineral is zinc oxide (ZnO), a non-porous solid devoid of any drug loading capacity, that has long been used to treat cutaneous wounds and pathological skin conditions (Lansdown et al., 2007). In contrast to ZnO, versatile drug loading, release and disintegration rate of porous Zeolitic Imidazolate Frameworks (ZIFs) can be designed leading to various envisioned biomedical applications (Maleki et al., 2020). The most prominent member of the ZIF family is ZIF-8, which is a polymorph of zinc(II)2-methylimidazolate and has a ligand-metal-ligand bonding angle similar to that of O-Si-O. In addition, ZIF-8 shares the crystal topology (SOD) with Sodalite, a naturally occurring zeolite of the sodium aluminum silicate framework family.

## Preparation Methods of MOFs Suitable for Term Laboratories and Their Key Properties

### Solution Precipitation Synthesis of ZIF-8 and Loading of Bioactive Agents

ZIF-8 is easily synthesized through simple precipitation reactions facilitated by mixing the ligand (2-methylimidazole) and zinc salt solution at room temperature (Park et al., 2006; Beyer et al., 2016, 2018b; Kulow et al., 2019). ZIF-8 can be engineered to acquire bioactivity beyond that of zinc ions through encapsulation of drugs. Admixtures of Dexamethasone (Sarkar et al., 2019) or non-steroidal anti-inflammatory drugs (Ho et al., 2020) to these precursor mixtures during precipitation reactions led to their firm entrapment within ZIF-8. ZIF-8 shows a sustained release behavior for small molecules up to several weeks (Liédana et al., 2012). An interesting aspect of ZIF-8 is the observation that it crystallizes around biomacromolecules in aqueous solution by a process termed biomimetic mineralization (Liang et al., 2015a,b; Gao et al., 2019). This approach has been used for delivery of insulin (Duan et al., 2018) and may provide opportunities for e.g., growth factor delivery.

### Mechanochemical Synthesis of MOFs and Potential Use as Filler in Polymer Composites

MOFs may also be synthesized mechanochemically (Stolar and Užareviæ, 2020) by mixing organic linkers and metal precursors in a small planetary ball-mill (Akhmetova et al., 2019), immediately yielding MOF powder in large quantities and short time. So far, the mechanochemical synthesis of calcium and strontium MOFs has been reported (Al-Terkawi et al., 2017), which is an excellent route to produce MOFs as bioactive fillers for e.g., polymeric biomaterials. However, mechanochemical syntheses routes for earth alkaline metal MOFs specifically for TERM applications have yet to be developed.

### Solvothermal Synthesis of CU-BTC

During solvo-thermal synthesis, two precursor solutions of organic ligands and metal ions are mixed and heated in a micro-autoclave beyond the boiling point of the solvent (Ni and Masel, 2006). Copper(II)1,3,5-benzenetricarboxylate (Cu-BTC), also known as HKUST-1 (Chui, 1999) is typically synthesized under solvo-thermal conditions. The porous structure of Cu-BTC makes the copper metal center accessible to solutes. This renders Cu-BTC an interesting catalyst for the conversion of blood borne nitrosothiols to continuously release nitric oxide (NO) (Harding and Reynolds, 2012). Nitric oxide is a key messenger molecule during inflammation (Sharma et al., 2007), platelet adhesion (Radomski et al., 1987), ECM deposition (Myers and Tanner, 1998), angiogenesis and other aspects of tissue healing and foreign body response (Schäffer et al., 1996). Beyond the intrinsic catalytic activity, constant release of copper ions is crucial for the biological activity of Cu-BTC. Copper ions are known to stimulate angiogenesis (Sen et al., 2002) and to increase extracellular matrix (ECM) deposition (Gérard et al., 2010). These properties together hold potential to benefit TERM applications of copper-based MOFs when correctly dosed for the intended application.

### Electrochemical MOF Coating With Potential Use in Sensors or Electrodes

Electro-deposition of MOFs (Campagnol et al., 2016) may be useful for coating of conductive, implantable electrodes and sensors especially with future applications in neuro-interface engineering.

## Applications of MOFs or MOF Based Biomaterials in Term

### MOFs for Orthopedic Implants

Most reports on use of MOFs in TERM applications focus on the improvement of structural bone implants. Indeed, a small but significant fraction of clinical implants fail due to an unfavorable, non-healing wound environment that may be aggravated by bacterial infection (Inacio et al., 2013).

#### Intrinsic Bioactivity of MOFs and Influence on Bone Tissue Biology

*In vitro*studies showed significantly improved corrosion resistance of magnesium alloys upon coating with bio-MOF-1, comprising of zinc ions and adenine as well as interconnections of 4,4-biphenyldicarboxylate linkers (Liu et al., 2019). Besides engineering corrosion resistance for better structural integrity, the ability to prevent bacterial infection is crucial for successful integration of bone implants. Sr-HA (Sr-substituted hydroxyapatite) with MOF74 has demonstrated excellent abilities to inhibit *S. Aureus* and *E. Coli* bacteria and simultaneously induced apoptosis in Saos-2 cancer cells while promoting proliferation and maturation of primary osteoblasts (Zhang et al., 2019).

The effect of different metal ions on bone mineralization and osteogenesis is long known and mechanistic insights have been obtained e.g., for zinc (Kwun et al., 2010) or magnesium (Zhang et al., 2016). Consequently, MOFs were shown to promote osteogenic differentiation *in vitro*, an indicator for a better osteointegration of bone implants. ZIF-8 coatings on titanium implants enhanced ALP activity, better extracellular matrix mineralization and upregulation of osteogenic gene expression in MG63 cells, while suppressing Streptococcus Mutans proliferation (Chen et al., 2017a). Beyond zinc and magnesium, Ca-Sr MOFs could upregulate the expression of ALP, whereas simultaneous release of Ca and Sr provided osteo-inductive signals (Joseph et al., 2019). Further, simultaneous delivery of calcium and strontium ions as well as bisphosphonate linkers was noted to allow better bone mineralization (Matlinska et al., 2019). In the context of regenerative medicine, Mg/HCOOH-MOF was investigated for the treatment of osteoarthritis. This MOF was shown to up-regulate the expression of OCN, Axin2 and down-regulate iNOS and IL-1β, indicating a potentially favorable effect to ease osteoarthritis (Li et al., 2020).

*In vivo* investigations were less frequently reported, indicating that the field of MOFs for orthopedic applications is just at the beginning of being explored. Mg/Zn-MOF74, was demonstrated to have low toxicity, intrinsic anti-inflammatory and antibacterial features, while improving early osteogenic promotion and angiogenesis *in vivo* (Zhu et al., 2020).

#### Acquired Bioactivity of MOFs Through Drug Loading for Enhanced Bone Regeneration

Acquired bioactivity refers to any additional biological effect that does not originate from the MOF itself, which can be acquired through encapsulation of drugs, ions or other bioactive agents. MOF-based drug carriers are interesting for orthopedic implant coatings due to their high drug encapsulation capacity with a negligible premature release (Tan et al., 2016).

*In vitro* studies showed that Poly-l-lactic acid (PLLA) scaffolds coated with copper loaded ZIF-8 promoted osteogenic differentiation as compared to pure PLLA scaffolds while preventing bacterial infection in the presence of Zn^2+^ and Cu^2+^ ions (Telgerd et al., 2019).

Beyond the release of ions, MOFs have unique sustained release abilities for small molecular weight drugs. A sustained release of dexamethasone for 4 weeks was achieved through integrating dexamethasone loaded ZIF-8 into cellulose-hydroxyapatite nanocomposites envisioned for load bearing orthopedic applications (Sarkar et al., 2019). A similar approach has led to silk fibroin-dexamethasone@zeolitic imidazolate framework-8-titanium (SF-DEX@ZIF-8-Ti) composite materials. MC3T3-E1 osteoblast precursor cells cultured on the SF-DEX@ZIF-8-Ti showed enhanced osteo-differentiation (Ran et al., 2018). Hence, current research indicates a good potential of MOFs to facilitate osteointegration of bone implants, thereby improving their clinical outcome. Examples for treating bone diseases include the release of naringin, which improves osseointegration and prevents bacterial infection (Yu et al., 2017), delivery of antitubercular drug for treatment of osteoarticular tuberculosis (Pei et al., 2018) and release of vancomycin for treatment of osteomyelitis (Karakeçili et al., 2019). The versatility of MOF-based drug carriers principally allow the design of elaborate drug release mechanisms tailored to specific triggers. However, only one study describes pH and Ca^2+^ ion dependent release of drugs into the bone environment using a Zr-MOFs capped with CP5-based pseudorotaxanes (Tan et al., 2016).

*In vivo*studies are less frequently reported. One study investigated implant materials modified with levofloxacin loaded ZIF-8 particles, that resulted in better attachment, proliferation, and differentiation of osteoblasts *in vitro*. Special focus was given on the pH-triggered release in slightly acidic environments resulting from inflammation and bacterial infection. The effectiveness of this implant material was confirmed in terms of osseointegration and antibacterial properties in a rat infected femur model (Tao et al., 2020).

**FIGURE 2:**
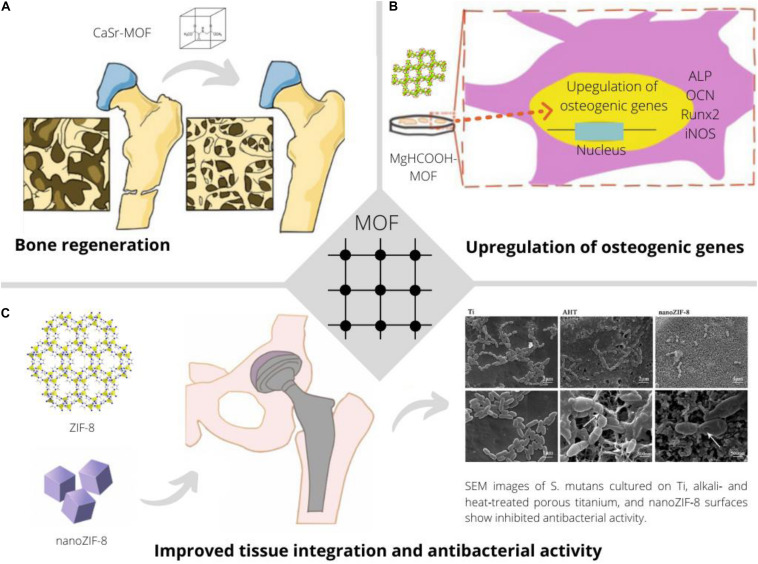
Application of MOFs to improve bone implants through continuous release of earth alkaline ions promoting bone regeneration and biomineralization (Joseph et al., 2019) **(A)**, facilitating osteo-induction and -integration through favorable upregulation of relevant genes in presence of specific MOFs (Li et al., 2020) **(B)** and providing improved tissue integration and an anti-bacterial effect (Chen et al., 2017a) **(C)**.

In conclusion, MOFs have been shown to be promising to enhance bone healing through intrinsic and/or acquired properties (Figure 2). While the here presented initial studies provide excellent perspectives for the use of MOFs for bone healing, more studies are needed toward their clinical applicability.

### MOFs for Cardio-Vascular Implants

Approximately 4% of the population in industrialized societies suffer from coronary artery disease (CAD) requiring intervention (Sanchis-Gomar et al., 2016). Traditionally, anti-thrombotic medication and small molecular weight nitric oxide donors acting as vasodilators are administrated to prevent artery occlusion. In more severe cases, drug-eluting stents are implanted into the artery to prevent occlusion permanently. Copper-based MOFs recently emerged as component in anti-thrombotic coatings for cardio-vascular implants.

*In vitro:* Cu-BTC was established to be an efficient catalyst for the conversion of blood borne s-nitroso-cysteine to nitric oxide and cysteine (Harding and Reynolds, 2012). More complex Cu-MOFs have subsequently been investigated for catalytic conversion of s-ntirosoglutathione (Neufeld et al., 2017b). This has led to works on MOF/polymer composite materials for nitric oxide release as suitable leads for novel implant materials (Harding and Reynolds, 2014; Neufeld et al., 2015, 2016, 2017a; Zang et al., 2020). In addition to these initial studies, Cu-BTC was directly grown on stent surfaces as SURMOF and investigated *in vitro* showing favorable hemocompatibility (Zhao et al., 2019).

Evaluating non-copper based MOFs for cardiovascular implants, a MIL-101 (Fe) poly(ε−caprolactone) composite was investigated as material for metal free stents, using the MOF as mechanical re-enforcement, sustained drug release vehicle and Magnetic Resonance Imaging (MRI) contrast agent (Hamideh et al., 2020). Further, few studies exist that address the interaction of MOFs with cardiomyocytes (Al-Ansari et al., 2020).

*In vivo,* Cu-BTC was shown effective as cardiovascular stent surface modification through coating together with polydopamine (Fan et al., 2019). This significantly reduced protein absorption, thrombus formation, platelet adhesion and an increased vasodilating effect, especially upon systemic injection of nitric oxide donors requiring catalytic degradation. Using a 5-(1H-tetrazol-5-yl)isophthalic acid based MOF with Ni^2+^ and K^+^ ions in another study prevented arrhythmia through reducing the sympathetic excitability in a rat model (Ding et al., 2020). Future studies will need to compare the effectiveness and safety of MOFs with that of clinically used materials.

### MOF-Based Biomaterials for Cutaneous Wound Care

The clinical healing outcome of chronic wounds arising from pressure ulcers or injuries of diabetic patients is often unsatisfactory, which motivates research for better cutaneous wound care material. In addition, achieving fast hemostasis and antibacterial properties is important for emergency care equipment and the potential role of MOFs for these two applications is elucidated in the following.

*In vitro* studies investigated polymer meshes with enclosed Cu-BTC (Zhang et al., 2020) and cellulose based biomaterials with the ability to release nitric oxide (Darder et al., 2020) as wound dressing. To ascribe increased mitochondrial respiration solely to nitric oxide, a titanium-based MOF devoid of intrinsic bioactivity and with controlled release of pre-loaded nitric oxide was proposed to be a copper free alternative (Pinto et al., 2020).

*In vivo*studies have shown the positive effect of Cu-BTC on skin healing using full-thickness splinted skin wounds in rodents. Dosage forms included folate stabilized Cu-BTC crystal suspensions (Xiao et al., 2018) and hydrogel compositions (Xiao et al., 2017).

Supplementation of zinc ions from ZIF-8 has shown to be similarly effective in a ZIF-8 hydrogel composite membrane (Yao et al., 2020) for wound healing. Combination of zinc and copper ions in MOFs that further encapsulated bioactive molecules promised to be most effective. Along this line of thought, the encapsulation of niacin loaded copper-/zinc MOFs within alginate shells has shown antibacterial, antioxidant and angiogenic properties, which resulted in significant improvement of wound closure in an infected full-thickness skin defect model (Chen et al., 2019).

Future studies are necessary to ascribe these effects to the synergistic potential of MOFs and not to the presence of its individual components upon dissolution.

### MOFs for Neuronal Tissue Engineering and Implants

Traumatic injuries to the nervous system result in dysfunctional regeneration and glial scaring in an inflammatory process leading to loss of nervous tissue. The consequences include physical paralysis of muscles, neurosensory or mental impairment. This has motivated the development of biomaterials for nervous tissue regeneration (Liu and Hsu, 2020), and MOFs may be suitable to further augment positive materials characteristic toward this goal.

*In vitro* studies focused on assessing the response of sensory neurons to nano-particulate MOFs by employing a neurite outgrowth assay, indicating the potential of a material to promote axonal outgrowth, thus nervous regeneration (Wuttke et al., 2017). The MOFs investigated included iron and chromium salts of trimesic acid (MIL-100) and Zirconium fumarate MOFs. These MOFs did not show reduction in neurite outgrowth at a concentration of 100 μg/ml, indicating their suitability for drug delivery applications. In line with the thought that MOFs are compatible with neuronal biology, treatment of neuronal stem cells with a cobalt MOF having 3,3’,5,5’ -azobenzenetetracarboxylic acid as linker was envisioned to benefit therapies for spinal cord injury. This was due to molecular docking studies revealing potential binding of the MOF with the trkA receptor, which led to activation of PI3K/Akt signaling and enhanced nerve growth factor (NGF) production *in vitro.* NGF is responsible for neuron growth and survival (Cao et al., 2020).

MOF based drug delivery vehicles include cationic MOF-74-Fe(III) with low cytotoxicity on PC12 neuron cells and high drug loading due to electrostatic accumulation (Hu et al., 2014) of ibuprofen, which is known to reduce neuroinflammation (Wixey et al., 2019) and is passively released from the MOF. An example for actively triggered dopamine release by magnetophoretic means was demonstrated using MIL-88A (iron(III) fumarate) encapsulated iron oxide particles (Pinna et al., 2018). A drug delivery mechanism that is triggered upon synaptic transmission can be achieved using adenosine triphosphate (ATP) responsive release of drugs. Here, a MOF based on Zr^4+^ ions and amino-triphenyldicarboxylic acid was capped with aptamers specific for ATP, leading to an un-locking effect upon binding and drug release (Chen et al., 2017b). These three examples of passive-, triggered- and targeted drug release show the versatility of MOF based drug delivery for neurobiological applications. Systemic injection requires MOF particles to cross the blood-brain barrier. This ability was assessed for e.g., ZIF-8 particles through crossing an hCMEC/D3 brain endothelial cell membrane (Chiacchia et al., 2015). Future studies in this direction would benefit from microfluidic vascular transport models for nanoparticles (Ho et al., 2017) involving pericytes to better emulate the blood-brain barrier and to study the biological response of further cell types in inflammation and healing (Beyer et al., 2018a; Blocki et al., 2018).

*In vivo*studies reported the use of a Cu(II) MOF in a mixed linker approach with 5-methylisophthalic acid and 1,3-bis(5,6-dimethylbenzimidazol-1-yl)propane (Tian and Tian, 2020). This MOF inhibited dopaminergic neurons, decreased expression of dopamine reporters, decreased dopamine release, reduced apoptosis and increased Wnt1-Nkx2.2 activation. The MOF was presumably injected on a systemic level into LgDel ± mice and biological effects were observed in a dose dependent manner up to 5 mg/kg body weight. No comparison of the biological effect upon injection of the MOF or a solution containing only the ligand was made.

A very inspiring study used MIL-100 (Fe) as a drug carrier for siSOX9 and retinoic acid in combination with co-encapsulated ceria based nanozymes to reduce oxidative stress (Yu et al., 2020). This complex drug formulation was incubated with neuronal stem cells (NSCs) isolated from newborn mice prior to NSC transplantation at sites of lesions. The co-release of soSOX9 and retinoic acid promoted neuro-differentiation and reduced oxidative stress prevented newborn neurons from apoptosis. The studies were carried out with 11-month old APPswe/PS1M146V/TauP301L triple transgenic mice, a model for Alzheimer’s disease. The positive effect of the MOF based drug combination was shown through cognitive improvements in a Morris Water Maze test and histopathological improvements.

These inspiring *in vitro* and *in vivo* works have highlighted a clear potential of MOFs to treat neurological disorders. Very few macroscopic biomaterials have been developed along this thought and future studies should include evaluating the opportunity to improve implant-nervous tissue interfaces with these materials.

## Conclusion and Outlook

The presented *in vitro* and *in vivo* data are impressive in terms of the strong biological response that MOFs elicit toward a positive healing outcome in various tissues and applications. However, more efforts are required to understand the basic dose dependent response of individual cell types and tissues to different MOFs. In addition, concerns about the safety of some organic linkers of MOFs need to be eliminated or alternative linkers considered save for clinical applications must be shown effective in yielding MOFs with similar properties. This is essential before MOF based biomaterials for TERM applications will commence improving healing outcomes in the treatment of human patients.

## Author Contributions

MS reviewed literature related to orthopedic implants and cutaneous wound care and designed the figures. JR reviewed literature related to neuronal TERM applications. XL reviewed literature related to nitric oxide producing biomaterials. JM reviewed literature related to MOF & polymer composite materials. AB and SB wrote the manuscript. SB guided the review process. All authors contributed to the article and approved the submitted version.

## Conflict of Interest

The authors declare that the research was conducted in the absence of any commercial or financial relationships that could be construed as a potential conflict of interest.
